# Data coverage of a cancer registry in southern Iran before and after implementation of a population-based reporting system: a 10-year trend study

**DOI:** 10.1186/1472-6963-13-169

**Published:** 2013-05-06

**Authors:** Kamran Bagheri Lankarani, Zahra Khosravizadegan, Abbas Rezaianzadeh, Behnam Honarvar, Mohsen Moghadami, Hossein Faramarzi, Mojtaba Mahmoodi, Mahin Farahmand, Seyed Masoom Masoompour, Bahman Nazemzadegan

**Affiliations:** 1 Health Policy Research Center, Shiraz University of Medical Sciences, 7134845794 Shiraz, Iran; 2 Department of Cancer Surveillance, Deputy of Health, Shiraz University of Medical Sciences, Shiraz, 7134814336 , Iran; 3 Research center for health sciences, shiraz university of medical sciences, shiraz, Iran; 4 HIV/AIDS Research Center, Shiraz University of Medical Sciences, Shiraz, 7134814336, Iran; 5 Shiraz University of Medical Sciences, Shiraz, 7134814336, Iran

**Keywords:** Cancer registry program, Trend, Completeness of coverage, Pathology-based, Population-based, Iran

## Abstract

**Background:**

Cancer registries help to decrease the burden of cancers by collecting accurate and complete data. We aimed to measure the completeness of coverage of information recorded between 2000 and 2009 in a cancer registry program in Fars province, southern Iran.

**Methods:**

The cancer registry program run by Shiraz University of Medical Sciences was investigated in two periods: pathology-based data from 2000 to 2007 and population-based data from 2007 to 2009. Completeness of yearly coverage was measured as the number of reported cases of cancer in each year divided by estimated cases based on 107.3 new cases per 100 000 individuals. The percentage of complete data registration (patient’s name, age, gender, address, phone number and father’s name) and correct cancer encoding was calculated for each year and compared to the maximum acceptable error rate for each item.

**Results:**

A total of 29 277 non-duplicate cancer records were studied. Completeness of coverage varied from 22.68% in 2000 to 118.7% in 2008. Deficiencies in patients’ demographic data were highest for name in 2002 (0.09%), age in 2006 (2.36%), gender in 2001 (0.06%) and father’s name in 2001 (52.5%). Incomplete address (99.7%) and missing phone number (100%) were most frequent in 2000, and deficiencies in encoding information were highest in 2008 (6.36%).

**Conclusions:**

The cancer registry program in Fars province (southern Iran) was considered satisfactory in terms of completeness of coverage and information about age. However, it was deficient in recording patients’ phone number and address, and father’s name. The error level for cancer encoding was unacceptably high. Enhancing hardware and software resources, education and motivation in all public and private sectors involved in the cancer registry program, and greater attention to epidemiological research are needed to increase the quality of the cancer registry program, including its completeness.

## Background

Cancer, which accounted for 13% of all global deaths in 2008, is predicted to become an increasingly important cause of morbidity and mortality in the next few decades in all regions of the world. The estimated percentage increase in cancer incidence by 2030 (21.4 million, including 13.1 million deaths) compared to 2008 (12.7 million, including 7.6 million deaths) will be greater in low- (82%) and lower-middle-income countries (70%) than in upper-middle- (58%) and high-income countries (40%) [1].

Iran, with a population of approximately 74 million, has an age-standardized death rate from cancer of 112.7 per 100 000 for males and 69.8 per 100 000 for females [1]. Cancer is the third leading cause of death in this country, [2] and accounted for 12% of all deaths [1] and 30 000 deaths among all ages in 2008 [2]. Moreover, it is estimated that more than 70 000 new cases of cancer occur in Iran annually [2]. In Fars province (southern Iran) the cancer-related age-standardized death rate in 1998–2002 was 64.5 per 100 000 for men and 55.5 per 100 000 for women [3].

Cancer registries, which originated in the first half of the twentieth century, have expanded in the last 20 years [4] and have become the main source of epidemiological information for all sectors involved in fighting cancer at the local to international level [5]. These registries systematically collect information about cancer burden by recording the incidence, prevalence, mortality and survival for different cancers [5,6]. Their role has expanded into the planning and evaluation of cancer treatments and screening programs, public health planning and patient care improvement [4,7-10]. The etiology, biology and impact of interventions to control cancers can also be studied through a variety of epidemiologic methods with data from cancer registries [8,11]. Projections about future needs for material and manpower resources can be made on the basis of data collected by registries [8].

The most comprehensive morbidity data for cancer are available from population- or hospital-based cancer registries, [1] but only population-based cancer registries can provide an unbiased description of cancer profiles in different populations [1]. However, only 36% of all countries have a national population-based registry [1].

The cancer registry system managed by Shiraz University of Medical Sciences was initially pathology-based from 2000 to 2007. Since 2007, the cancer registry system has become population-based. The system collects data on cancer cases from both hospital and nonhospital resources in a confidential manner. In its more than 10 years of operation, the registry has undergone qualitative and quantitative changes in the staff who collect the data, the centers reporting cancer cases and the software.

A crucial factor concerning the utility of cancer registries is data quality in terms of comparability, validity, timeliness and completeness [12,13]. The comparability of cancer data can be established through a comprehensive review of the registration procedures. Validity can be examined with numerical indices that permit comparisons across time with other registries or within a registry, or comparisons of specified subsets of cases. At present there are no international guidelines for timeliness, although some organizations have proposed specific standards for the abstraction and reporting of registries [12]. The completeness of cancer registry data, i.e. the extent to which all of the incident cancers occurring in the population are included in the registry database, is an extremely important attribute of a cancer registry. Only a high degree of completeness in case-finding procedures will ensure that cancer incidence rates and survival proportions are close to their true value [14]. The complete coverage of all cases is the key criterion for data quality [9].

### Objectives

This study was designed to document and evaluate the 10-year trends in completeness of coverage and completeness of recorded demographic data and encoding information in the Fars province cancer registry program. Our ultimate aim was to detect possible deficiencies and suggest the interventions needed to overcome them.

## Methods

The data source for this cross-sectional study was all cancer-related data recorded from 2000 to 2009 at a cancer surveillance center affiliated with Shiraz University Medical Sciences, southern Iran. To determine completeness of coverage per year, the number of new cancers was divided by the expected number of total cancers in that year in the target population. The expected incidence rate of cancer (excluding non-melanoma skin cancers) for Iran was estimated by the World Health Organization as 107.3 cases per 100 000 individuals [15]. The 10-year trend for completeness of coverage was determined from the actual registry data and compared the expected incidence rate.

To measure completeness of the data in the cancer registry, the percentage rate of deficiencies in identity (first name, last name and father’s name), demographic information (gender, age and area of residence) and encoding were calculated separately for each year and then compared to other years as well as to the maximum acceptable error rate established in the guidelines of the cancer registry program for each item. The maximum acceptable error rate was 5% for deficiencies in father’s name, demographic information and cancer encoding. The maximum acceptable error rate was 0.5% for deficiencies in first name, last name and gender. The data were entered into a Microsoft Office Excel 2007 spreadsheet and used to generate bar and linear graphs with this software.

The confidentiality of all data was ensured in all stages of the study from extraction of the data from the cancer registry to analysis, reporting and preserving the backup data. The Ethics Committee of the Health Policy Research Center affiliated with Shiraz University of Medical Sciences approved this study on the basis of the protocol described above.

## Results

**Figure 1:**
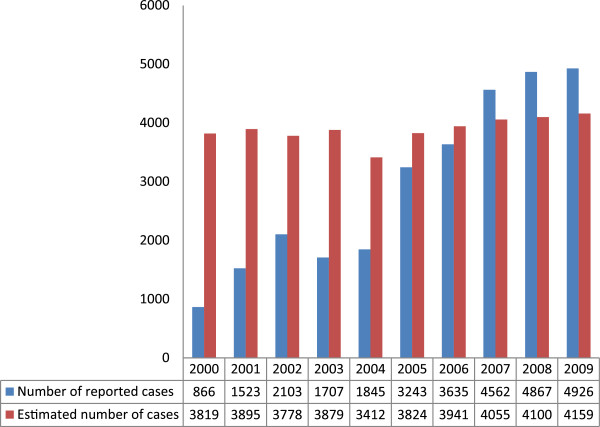
Number of reported and estimated cases of all cancers in 2000–2009 in Fars province, southern Iran.

**Figure 2:**
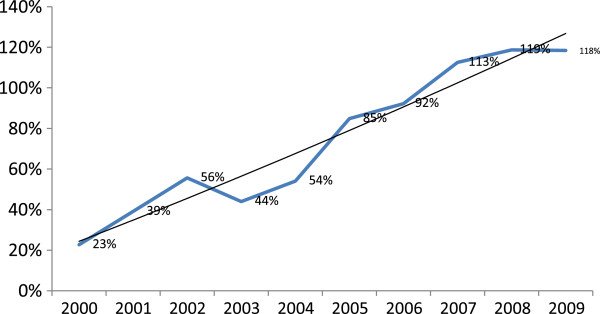
Trend in completeness of data coverage in 2000–2009 in the cancer registry program in Fars province, southern Iran.

A total of 29 277 nonduplicate cancer records were studied. Figure 1 shows that the number of cancer cases increased from 866 in 2000 to 4926 in 2009. Completeness of coverage also showed an overall increase from 22.68% in 2000 to a maximum of 118.7% in 2008. However, there was a decrease of about 11.5% in 2003 and a decrease of about 1.5% in 2004 compared to 2002, and a small decrease of 0.3% in 2009 compared to 2008 (Figure 2).

**Figure 3:**
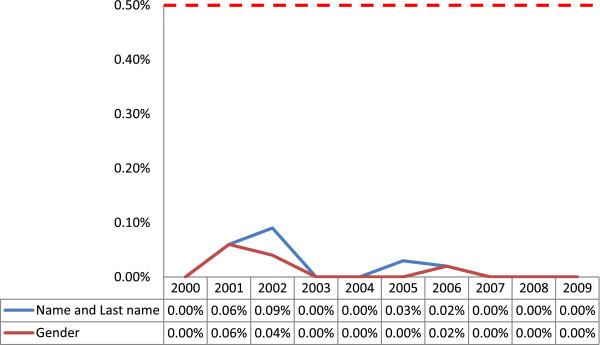
**Trend in incompleteness of data coverage for name and gender in 2000–2009 in the cancer registry program in Fars province, southern Iran. **The horizontal dotted axis shows the 0.5% level of acceptable error.

The rate of deficiencies in the first and last name showed a heterogeneous pattern. There were no deficiencies in 2000, 2003, 2004, 2007, 2008 and 2009. The rate of deficiencies was 0.06% in 2001, 0.09% in 2002, 0.03% in 2005 and 0.02% in 2006 (Figure 3). The trend in incomplete information about gender was also heterogeneous, with no deficiencies in 2000, 2003, 2004, 2005, 2007, 2008 and 2009, but rates of 0.06% in 2001, 0.04% in 2002 and 0.02% in 2006 (Figure 3). All of the deficiency rates in name or gender information were below the acceptable error level error of 0.5% (Figure 3).

**Figure 4:**
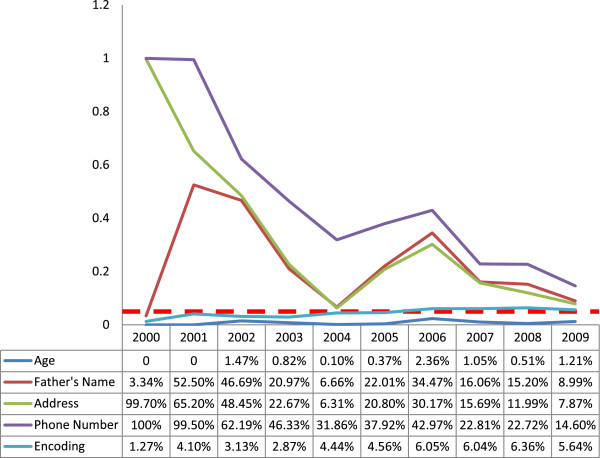
**Trend in incompleteness of data coverage for demographic, information, address and encoding in 2000–2009 in the cancer registry program in Fars province, southern Iran. **The horizontal dotted axis shows the 5% level of acceptable error.

The rates for incomplete data regarding patients’ age showed little variation from 2000 to 2009. The error rate was zero in 2000 and 2001 and highest in 2006 (2.36%); however, even this highest rate was below the 5% threshold for acceptable error rate (Figure 4).

Deficiencies in the father’s name were lowest in 2000 (3.34%) and highest in 2001 (52.5%). There was no uniform pattern from 2001 to 2006, but thereafter there was a downward trend until 2009. In all years except 2000, the error rate was above acceptable 5% limit; however, after 2006 the gap between real and expected error rates became narrower, decreasing to about 4% in 2009 (Figure 4).

Improvements in address information showed an optimal trend, from only 0.3% complete data in 2000 to 93.69% in 2004. About 20% of address entries in 2005 and 30% in 2006 were incomplete. From 2006 to 2009 the difference between the real and maximum acceptable error rate decreased; however, by the end of the study period in 2009 the error rate (7.87%) remained above the 5% threshold (Figure 4).

Of all variables analyzed here, phone numbers had the highest error rates. In 2000 and 2001, phone numbers were rarely recorded. In 2003 this information was missing for about half of the patients in the registry, and in 2004 and 2005 it was missing in about one-third of the cases. As Figure 4 shows, this error rate showed a downward trend between 2006 (44.97%) and 2009 (14.6%), but still remained above the acceptable 5% error rate at the end of the study period.

The percentage rate of encoding errors was lowest in 2000 (1.27%) and was below 5% (acceptable error rate) between 2000 and 2005. However, the frequency of errors surpassed the acceptable error rate in 2006 to 2009, although only by 0.64% in the final year of the study period (5.64%) (Figure 4).

## Discussion

Completeness of coverage of cancer-related data in the cancer registry program in Fars province, southern Iran, showed an increasing trend from 2000 to 2009, and was above 100% during the last three years of the study period. There are two possible explanations for the near-100% coverage in the last three years of the study period. One is the change in reporting resources from only pathology centers between 2000 and 2007 to pathology- and population-based resources from 2007 on. This resulted in the capture of non pathology-based cases, i.e. clinical reports and death records. The number of reported cases (numerator) thus increased compared to cases estimated on the basis of pathology-based cases (denominator). The second explanation may be the actual change in cancer incidence in Iran due to changes in life style and predisposing factors, a process that necessitates recalculation of the estimated numbers of cancer in this country. However, these two possibilities will require additional research to confirm.

Throughout the 10-year study period, data incompleteness for name and gender of the patients in the registry were far below the acceptable error rate of 0.5%, and the error rate was zero during the last three years. However, the data for phone number, address and father’s name showed the highest rate of deficiencies, which remained over the acceptable error rate of 5% in all 10 years, with the exception of father’s name in 2000. The gap between actual and acceptable error rates for each item showed a decreasing trend toward 2009, when the error rate was lowest. Encoding showed an incompleteness rate around 5% between 2004 and 2009, but remained above the 5% acceptable error rate from 2006 to 2009. Errors in age were below half of the acceptable error rate of 5%, and were as low as 1% during the last three years of the study period.

Improvements in the completeness of converge in our regional cancer registry program may have resulted from better communication with reporting centers such as pathology laboratories and clinical centers, which were sent directives and guidelines regarding the registry as well as information regarding the obligation to report cancer cases. Similar improvements have also been seen in Iran’s national cancer registry, which showed a remarkable increase in completeness of coverage from 1999 (29%) to 2008 (92.85%) [16].

A study in Kampala, Uganda found that the completeness of registration of diagnosed cancer cases was 89.6% (95% CI 87.0-91.7). Completeness varied with age (better for patients less than 30 years old) and cancer site, and cases with a histology report were more likely to be registered than those without one [17]. A study in the French Maritimes Alps district found that completeness of their registry was 92.2% (95% CI: 91.5-93.0%) for breast and colorectal cancer [18]. Overall completeness of a cancer registry in Estonia was 90.8%, although the rate varied by cancer site [19].

Completeness of reporting for ovarian cancer from one health region in Norway during the 10-year period from 1987 to 1996 was 99.6% [20]. An evaluation of a cancer registry program in Karachi, Pakistan showed a number of defects at the beginning in 1995 but a marked increase in completeness and accuracy of the data in 2000 [21].

In Fars province, reporting of the patient’s full name in the cancer registry showed considerable improvement, especially after 2007, as also occurred in the national registry: the error rate was zero or close to zero in most years. Therefore, deficiencies in this information remained negligible. This finding shows that reporting centers have been well informed about the importance of obtaining complete information on each patient’s identity [16].

In the cancer registry analyzed here, the percentage of deficiencies in gender-related data was quite small and consistently below the threshold of acceptable error throughout the study period.

The performance of the provincial registry we analyzed in terms of completeness of age-related data improved during the 10-year study period, and a similar pattern was also reported for national registry [16]. Increasing resources for case reporting during the period from 2000 to 2009 in our region, especially in last three years of this period, resulted in more complete recording of the father’s name in provincial registry, as was also found for the national registry [16].

At the beginning of the registry program, the patients’ address was not recorded in detail in a systematic manner. Although the rate of deficiencies decreased as a result of staff training at reporting centers, the error rate remained above our acceptable maximum level. This finding confirms the need for planning in order to improve the completeness of address-related information. In the national cancer registry, errors in this item decreased from 66.5% in 1999 to 21.4% in 2008 [16].

The completeness of information about cancer encoding fluctuated throughout the study period. After 2007, when the data were collected with both pathology and population-based system, encoding errors and cases of cancer of unknown origin and undefined codes have become more frequent. However, Iran’s national level cancer registry showed a decrease in encoding deficiencies [16]. A 10-year study of a cancer registry in Norway reported a coding error rate of 2% [20].

Cancer registries need to ensure the quality of their data. Earlier studies showed that stringent quality assurance procedures can be used to achieve perfect accuracy, completeness, timeliness and confidentiality [20,22]. However, these aims can only be achieved through planning, regulating, promoting and applying procedural rules, and by supervising the correct implementation of quality assurance measures. Such efforts in Iran, as in other Asian countries, [23] are hampered by deficiencies in expertise and resources, infrastructural weaknesses, and a lack of epidemiology-based studies of case registry performance. Because the burden of cancer is expected to increase, strengthening the hardware and software resources needed for cancer registry programs will play an essential role in achieving and maintaining high standards in cancer case registration. Increases in the number of trained staff responsible for data collection will also be needed. As other studies have emphasized, it is important to educate all cancer registry data providers including pathology departments as well as population-based centers, including clinical centers and centers involved in the death registry program, about the importance of reporting accurate, timely and complete information [8,22,24].

The feedback provided by data registrars plays an important role in efforts to improve the software used in cancer registry programs. One indirect method to investigate data completeness is to measure indicators such as the ratios of and overlap among cases based on clinical and pathological reports and the death certificate only [25]. However, the reporting system used for the Fars province cancer registry program was pathology-based until 2007, so these indicators could not be measured or compared for the years prior to 2007.

A source of potentially useful information in cancer registries is data about the patient’s ethnicity. The population of Fars province, like the population of Iran, comprises several ethnicities, and data about the distribution of cancers among these different groups can be valuable for research on the etiological factors involved in different types of cancer. A further way to enhance the completeness and validity of the data in such registries is to include ongoing research activities regarding different aspects of cancer registry programs, as suggested in an earlier Danish analysis [26].

### Limitations

Our study had some limitations. We measured the completeness of data for all cancers as a whole. Different studies have shown that this index can differ significantly among cancers according to site [18,20] and age [18]. Furthermore, we could not determine how much of the increase in reported cancer cases after 2005 and especially after 2007 was due to an actual increase in the cancer incidence in our region, and how much was due to the change from pathology centers only to both pathology-based and population-based reporting systems.

## Conclusions

In the cancer registry used in Fars province, southern Iran, the completeness of coverage and information were acceptable with regard to gender, first name, last name and age. The error rates for these data were below the maximum acceptable threshold. However, with regard to father’s name and cancer encoding, the performance of the registry failed to meet our standard for maximum acceptable error rate. Currently about 25% of the primary data in the cancer registry is duplicated in any given year, and considerable time is needed to match and delete the repeated data, as is done annually in collaboration with the national cancer registry. Information for the patients’ national identification number was also inadequate. Improving the quality of the cancer registry program in Iran will require funding for appropriate infrastructure, enhanced hardware and software resources, and increased expert staffing. Education and training are needed for current staff, together with ways to encourage and motivate public and private sectors of the cancer registry program (e.g. physicians, pathology laboratories and surgical services) to complete the cancer case registry forms accurately and remit them to the appropriate center in a timely fashion. Additional improvements could be achieved by integrating ongoing research with epidemiological studies in the cancer registry program.

## Competing interests

The authors declare that they have no competing interests.

## Authors’ contributions

KBL made substantive intellectual contributions to study and participated in the conception, design and critical review of the manuscript. ZK was involved in conducting the study, data collection and analysis. AR played a major role in developing the study design and writing the manuscript. BH is the corresponding author and substantially revised and rewrote the draft and final manuscript. MM, HF, MM, MF and SMM all contributed to the study conception, data collection and critical review of the manuscript. All authors approved the final manuscript.

## Pre-publication history

The pre-publication history for this paper can be accessed here:

http://www.biomedcentral.com/1472-6963/13/169/prepub
